# In Silico Analysis of a Highly Mutated Gene in Cancer Provides Insight into Abnormal mRNA Splicing: Splicing Factor 3B Subunit 1^K700E^ Mutant

**DOI:** 10.3390/biom10050680

**Published:** 2020-04-28

**Authors:** Asmaa Samy, Baris Ethem Suzek, Mehmet Kemal Ozdemir, Ozge Sensoy

**Affiliations:** 1 The Graduate School of Engineering and Natural Science, Istanbul Medipol University, 34810 Istanbul, Turkey; 2 Department of Computer Engineering, Muğla Sıtkı Koçman University, 48000 Muğla, Turkey; 3 The School of Engineering and Natural Science, Istanbul Medipol University, 34810 Istanbul, Turkey; 4 Regenerative and Restorative Medicine Research Center (REMER), Istanbul Medipol University, 34810 Istanbul, Turkey

**Keywords:** cancer, somatic mutations, aberrant RNA splicing, SF3B1, missense mutations, molecular dynamics simulations

## Abstract

Cancer is the second leading cause of death worldwide. The etiology of the disease has remained elusive, but mutations causing aberrant RNA splicing have been considered one of the significant factors in various cancer types. The association of aberrant RNA splicing with drug/therapy resistance further increases the importance of these mutations. In this work, the impact of the splicing factor 3B subunit 1 (SF3B1) K700E mutation, a highly prevalent mutation in various cancer types, is investigated through molecular dynamics simulations. Based on our results, K700E mutation increases flexibility of the mutant SF3B1. Consequently, this mutation leads to i) disruption of interaction of pre-mRNA with SF3B1 and p14, thus preventing proper alignment of mRNA and causing usage of abnormal 3’ splice site, and ii) disruption of communication in critical regions participating in interactions with other proteins in pre-mRNA splicing machinery. We anticipate that this study enhances our understanding of the mechanism of functional abnormalities associated with splicing machinery, thereby, increasing possibility for designing effective therapies to combat cancer at an earlier stage.

## 1. Introduction

Cancer is considered a complex disease caused by a combination of predisposing genetic variants and environmental factors [1]. Moreover, the genetic background of the mutation and/or epigenetic alterations further increase the risk of development of the disease. The somatic mutations affecting the “pre-mRNA splicing machinery” are particularly critical as they cause aberrant splicing [2,3,4] that leads to formation of proteins with altered functions. Various studies have shown that these altered proteins are dominantly involved in processes such as tumor metabolism, apoptosis, cell cycle control, invasion, metastasis, and angiogenesis [5,6].

Processing of an intron-containing pre-mRNA consists of three steps: i) intron excision, ii) 3’-end processing, and iii) transportation of mature transcripts from nucleus into the cytoplasm [7]. The splicing process is carried out by a complex ribonucleoprotein (RNP) called spliceosome [8,9] which is composed of five small nuclear ribonucleoproteins (snRNPs) (U1, U2, U4, U5, and U6) along with other related proteins [10]. SF3b complex is the major component of U2 small nuclear ribonucleoprotein (U2snRNP) and it is responsible for reinforcing the interaction between U2 snRNA and the branch site on pre-mRNA in the first transesterification reaction of splicing process [11,12,13,14].

SF3b is composed of seven subunits: SF3B1, SF3B2, SF3B3, SF3B4, SF3B5, SF3B6, and SF3B7 [15]. Among them, splicing factor 3B subunit 1 (SF3B1) is the largest component of the SF3b complex [16,17] and it is composed of N-terminal helix-loop-helix domain (NTD) and C-terminal of 20 HEAT-repeat domain (HD) [15,18]. SF3B1 interacts directly with SF3B3, SF3B7, and SF3B5, all of which facilitate its binding to pre-mRNA. In addition, SF3B1 also interacts with SF3B6 (also known as p14) [15,19,20], two of which form a complex with U2 auxiliary factor, U2AF65, to recognize and select the branch splicing site properly [12,14,20]. In particular, the superhelical heat domain of SF3B1 provides a platform for effective binding of interacting partners and/or RNAs to the complex [21].

The point mutations occurring in the heat domain of SF3B1 promote usage of alternative branch points, hence abnormal 3’ splice sites. Consequently, this causes either (partial or complete) intron retention or excision of an exon in pre-mRNA. Indeed, it has been shown that SF3B1 mutations increased the number of transcripts with retained introns as seen in myelodysplastic syndrome [22] or they caused production of transcripts that included a shorter copy of the exon as seen in chronic lymphocytic leukemia [3]. In another study, it has been shown that mutations affecting SF3B1 gene caused alternative splicing of key genes such as UQCC, F8, ABCC5, and GUSBP11 in ER (estrogen receptor)-positive breast cancer [23]. The abnormal transcripts follow two possible pathways: 1) translation of mRNA into proteins with truncated or missing domain(s) which consequently leads to change, loss or gain of function of the protein [24] and 2) degradation by nonsense-mediated mRNA decay (NMD) mechanism which leads to a reduction in the proteome content of the cell [25]. Therefore, understanding the molecular mechanism behind aberrant splicing may help in preventing cancer development or progression; however, the number of related studies is scarce. Recently, Borisek et al. studied the impact of K335E, L378V, and N295D mutations on Hsh155, which is the yeast homolog of human SF3B1, using atomistic molecular dynamics to investigate the mechanism underlying constitutive/alternative/aberrant splicing [26]. In another recent study, Borisek et al. investigated the impact of splicing modulators such as pladienolides, herboxidienes, and spliceostatins, which have been shown to exert anti-tumor activity, using mutant Y36C and R38C of PHF5A as well as R1074H of SF3B1, two of which are the components of SF3b [27].

In this study, we utilized computational biophysics tools to investigate the impact of K700E mutation on structure and dynamics of SF3B1. Comparative analysis of molecular dynamics trajectories of wild type and mutant protein revealed that the mutation i) increased the flexibility of the protein, thus preventing proper alignment of pre-mRNA within the splicing machinery, ii) caused disruption of interactions between p14 and pre-mRNA. K700E mutation is shown to be associated with various cancer types including myelodysplastic syndrome (MDS), chronic lymphocyctic leukemia (CLL), uveal melanoma (UV), and pancreatic cancer [28]. Therefore, our results can provide insights into the molecular mechanism of aberrant splicing in various cancer types, highlighting the importance and general applicability of our work.

## 2. Results

### 2.1. SF3B1 Gene Has the Highest Number of Mutations in the Pre-mRNA Splicing Network

We identified a somatic mutation to further study, namely SF3B1${}^{K700E}$, factoring in two aspects; the associated gene’s centrality in pre-mRNA splicing machinery and the prevalence of the somatic mutations it carries. To this end, we constructed a pre-mRNA splicing network and superimposed the number of mutations obtained from latest version of COSMIC database on this network.

The network analysis revealed the top six genes with high centrality in the network which are SF3B1, splicing factor 3A subunit 2 (SF3A2), DEAH-Box helicase 15 (DHX15), U2 auxiliary factor 2 (U2AF2), pre-mRNA-processing factor 19 (PRPF19), and serine/arginine-rich splicing factor 1 (SRSF1). SF3A2 is the only component in the SF3a complex that recognizes the U2/BS duplex [18,29]. DHX15 is required to catalyze the disassembly of spliceosome complex after the completion of splicing and releasing mature mRNA to cytoplasm [30,31,32]. U2AF2 recognizes and binds to the polypyrimidine (Py) tract on pre-mRNA [33]. PRPF19 (also known as PRP19) interacts with other components of the NineTeen complex (NTC) that is essential for NTC stability [34], which is responsible for strengthening interactions between mRNA and snRNAs [35]. SRSF1 (also known as SF2/ASF) regulates pathways of mRNA metabolism such as pre-mRNA splicing [36], export [37], and translation [36]. The centrality metrics of SF3B1 including closeness, radiality, and eccentricity were equal to 0.58, 0.88, and 5, respectively which are slightly lower than the other five genes. However, SF3B1 was the most frequently mutated gene (associated with 34 highly pathogenic missense mutations) among other genes included in the constructed pre-mRNA splicing network. SF3A2, DHX15, U2AF2, PRPF19), and SRSF1 had 1, 13, 2, 6, and 2 highly pathogenic missense mutations, respectively.

**Figure 1:**
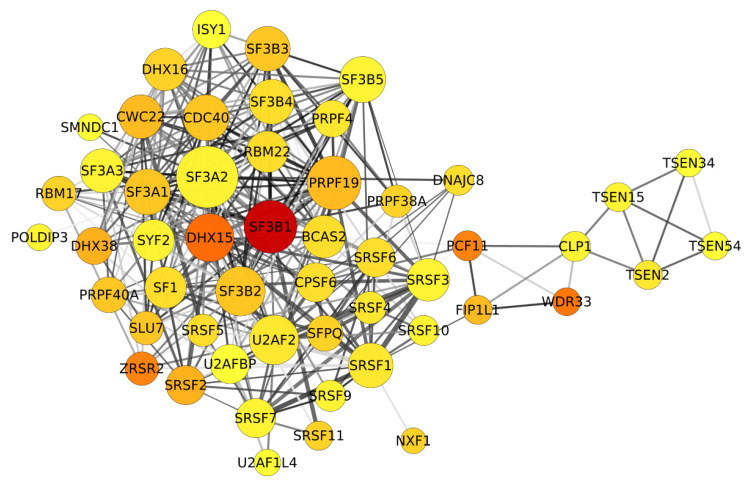
Depiction of the extracted network’s component. It consists of 54 nodes and 382 edges. In this component, nodes represent genes that are connected with 382 edges depending on different experimental data. Nodes’ color is mapped according to the number of associated mutations whereas nodes’ size is mapped according to their degree. An edge between every two nodes corresponds to their experimental interaction as explained in the methods section.

To maximize the general applicability of our study, we favored the prevalence of mutation over gene network centrality in deciding the mutation to further study. SF3B1 was a gene containing the highest number of mutations while having comparably high centrality in the constructed pre-mRNA splicing network to other genes as shown in Figure 1. Among the 34 highly pathogenic SF3B1 missense mutations, SF3B1 K700E mutation was the most prevalent mutation in various cancer types whose significance supported by previous experimental studies. Hence, we decided to study the impact of K700E mutation on the structure and dynamics of by means of MD simulations.

### 2.2. K700E Mutation Affects the Stability of SF3B1, Pre-mRNA, and p14

**Figure 2:**
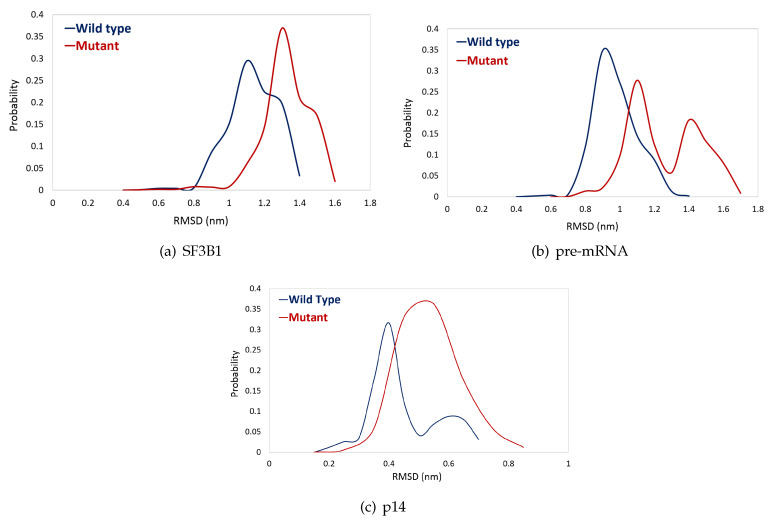
Probability plots of root mean square deviation (RMSD) distributions of (**a**) SF3B1${}^{wt}$ in blue vs. SF3B1${}^{K700E}$ in red. (**b**) pre-mRNA in the wild type structure in blue vs. mutant structure in red. (**c**) p14 in the wild type structure in blue vs. mutant structure in red.

K700E mutation is located in the heat domain of SF3B1 and is within the interaction distance from pre-mRNA (See Figure 8). The distance between the side chain of K700 and pre-mRNA is smaller than 0.20 nm. Therefore, we set out to investigate the impact of the mutation on the stability of pre-mRNA and also SF3B1 using trajectories obtained from MD simulations. The results showed that the mutation decreased the stability of pre-mRNA and SF3B1, which is evident from the right-shift in probability distributions of RMSD in the mutant system as shown in Figure 2a,b. We also did the same analysis for p14, which is shown to be involved in selection of the branch point on pre-mRNA, and observed a similar impact as well.

### 2.3. K700E Mutation Weakens Interactions Formed with Pre-mRNA

**Figure 3:**
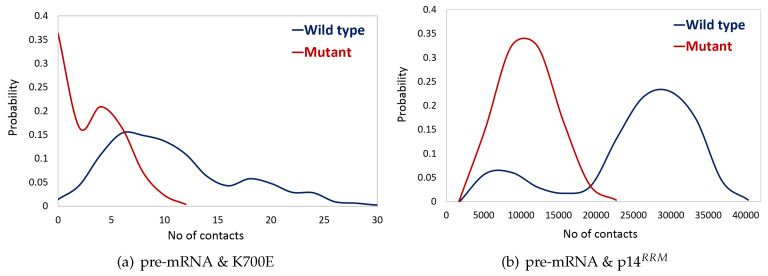
Probability plots of distributions of the number of contacts formed between (**a**) pre-mRNA and side chain of amino acid K700 in wild type shown in blue vs. E700 mutant shown in red, with cutoff distance ≤0.3 nm. (**b**) pre-mRNA and side chains of p14 residues (20–100) in wild type shown in blue vs. mutant shown in red, with cutoff distance ≤3 nm.

As shown in the above section, K700E mutation decreases stability of both SF3B1 and pre-mRNA, which presumably impacts interactions formed between the heat domain of SF3B1 and pre-mRNA. To test this, we calculated the number of contacts between K700 and pre-mRNA. The results showed that contact number was smaller in the mutant protein than in the wild type as shown in Figure 3a. Specifically, the number of contacts reached to zero after 250ns in the mutant protein. Presumably, this drastic reduction in the number of contacts has resulted from disruption of electrostatic interactions formed between pre-mRNA and K700. In the wild type, positively charged side chain of lysine “K700" attracts negatively charged phosphate backbone atoms of pre-mRNA [38], whereas negatively charged side chain of glutamic acid “E700" causes repulsion in the mutant system.

Additionally, we also calculated the number of interactions formed between pre-mRNA and p14, in particular, side chains of residues (20–100), as they are shown to be cross-linked to adenosine nucleotide at the branch point during splicing [29,39], hence known as RNA recognition motif (RRM) [20]. The results showed that the number of contacts formed between p14 and pre-mRNA decreased in the mutant protein as shown in Figure 3b.

### 2.4. K700E Mutation Impacts the Global Dynamics of SF3B1

**Figure 4:**
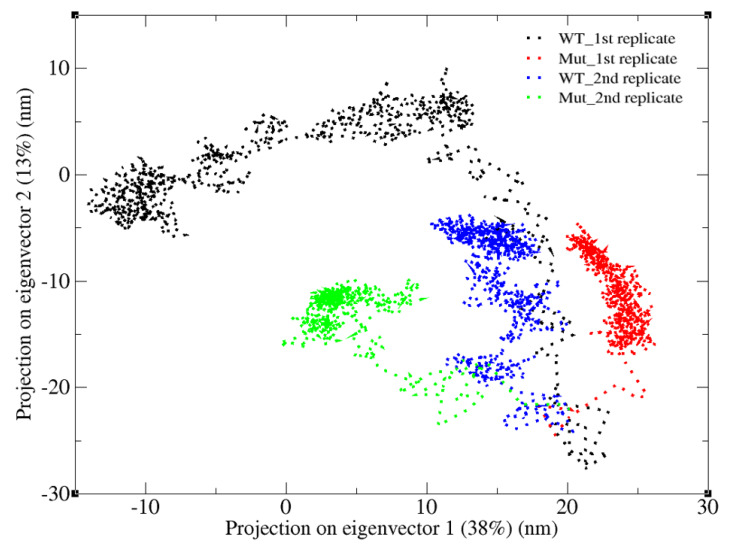
2D projection of SF3B1 trajectories for two replicates of the wild type protein (black and blue) and two replicates of the mutant system (red and green) along the first two eigenvectors of SF3B1${}^{K700}$ system.

As shown in the previous section, K700E mutation increased the flexibility of both SF3B1 and pre-mRNA. To further investigate the impact of the mutation on global dynamics of SF3B1, we performed essential dynamics analysis. To do so, we projected trajectories of both wild type and mutant SF3B1 systems along the first and the second eigenvectors as shown in Figure 4. With that, we could capture more than 50% of overall dynamics in these systems. The results showed that both replicates of the wild type system followed similar paths even though the first one sampled a wider space than the second one. Presumably, the protein in the second replicate might be trapped (See blue in Figure 4) in one of the many local energy minima present on the rugged potential energy surface. On the other hand, the two replicates followed completely different paths (See green and red color in Figure 4) in the mutant system which might be due to the increase in the protein flexibility upon mutation.

**Figure 5:**
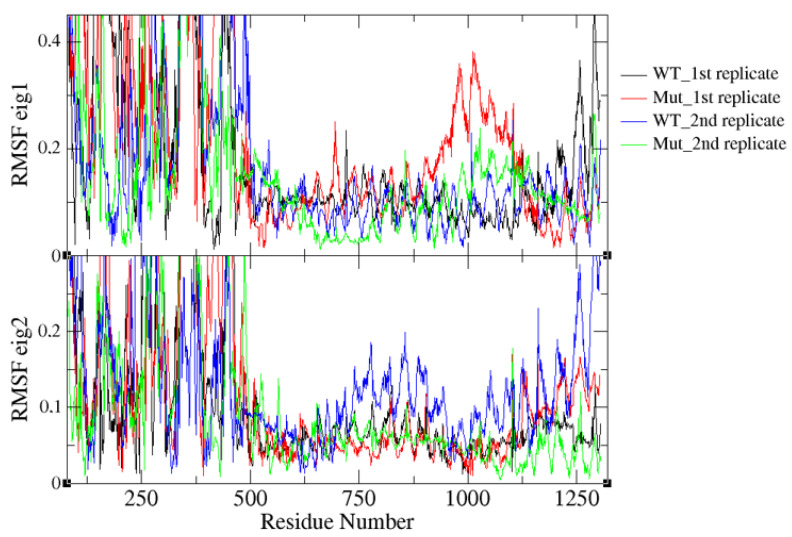
Root mean square fluctuation (RMSF) per Cα atoms of the first two eigenvectors of SF3B1 obtained for two replicates of the wild type protein (black and blue) and two replicates of the mutant system (red and green).

We further computed the RMSF values per Cα atoms of the protein along the first and the second eigenvectors of both wild type and mutant SF3B1 which cumulatively account for more than 50% of overall motion in the systems studied. The results showed that residues 900–1125 displayed relatively higher fluctuation in the first and the second replicate of the mutant system (See red and green color in the first eigenvector) as shown in Figure 5. On the other hand, we observed relatively higher fluctuation for residues 750–900 and 1125–1300 in the wild type system (See blue color in the second eigenvector); however, the contribution of these residues to the overall dynamics is smaller (15%) than did by the first eigenvector (37%).

### 2.5. K700E Mutation Changes the Correlation between Residues in SF3B1 Protein

**Figure 6:**
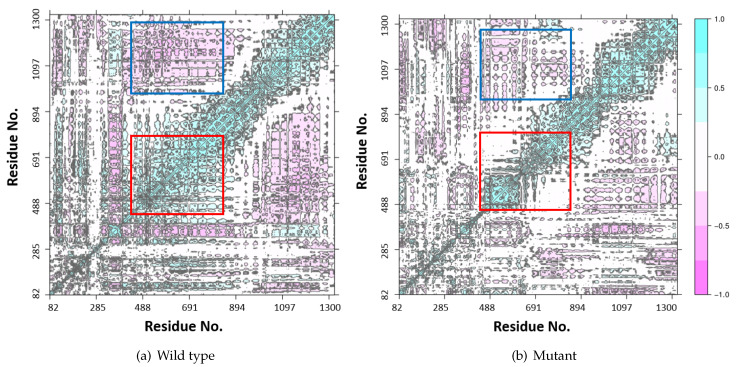
Dynamic cross correlation map for SF3B1 is shown in (**a**) for the wild type system. As can be seen, there is a high positive correlation within the region 455–832, which is colored in red, and a high negative correlation between two highlighted regions sequences as 455–832 in red and 932–1300 in blue. (**b**) For the mutant system, the positive correlation decreased drastically within the region 455–832, which is colored in red, and low negative correlation between the two regions 455–832 in red and 932–1300 in blue.

**Figure 7:**
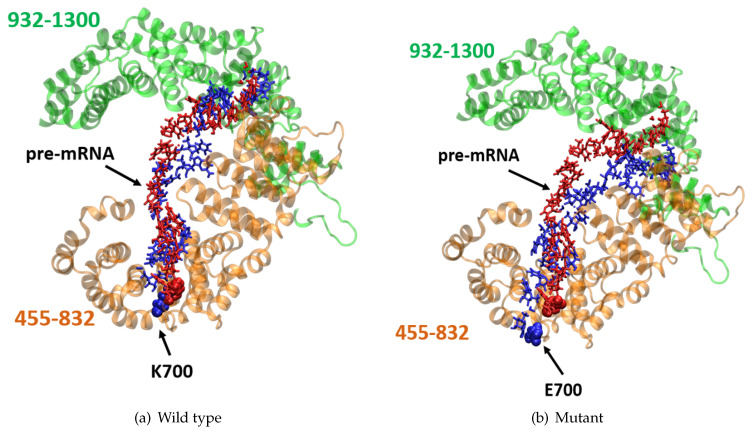
3D structure of the two highlighted regions surrounding pre-mRNA in wild type system (**a**) and mutant system (**b**). α helical region (455–832) in orange, α helical region (932–1300) in green, both of pre-mRNA and K700E residue are shown in red representing the initial frame of simulation and blue color representing the last frame. The structural image was created using VMD [40].

To investigate how the differences observed between global dynamics of wild type and mutant protein are reflected in the correlation patterns of SF3B1 residues, we made dynamic cross correlation analysis (Figure 6). As shown in Figure 7a it produced an NxN heatmap, where N corresponds to the number of Cα atoms in the protein. Moreover, each element in the matrix represents the degree of cross correlation, which is color-coded, between each atom. The negative and positive correlation is represented by pink and cyan, respectively. The results showed that the region that is made up of residues 455–832, which is indicated by red rectangle in Figure 7a, shows strong positive correlation in the wild type system. On the other hand, this correlation is lost in the mutant protein. Moreover, the negative correlation was seen between residues (455–832) and residues (932–1300), which is indicated by blue rectangle in Figure 7a, is also decreased in the mutant protein. Thereafter, we mapped the two regions, which are indicated by red and blue rectangles in Figure 7a, on the 3D structure of SF3B1 and showed that the two regions surround the pre-mRNA from its N- and C-terminus as shown in Figure 7a. To investigate how these regions interact with pre-mRNA throughout the trajectories, we aligned both wild type and mutant protein individually and examined the positioning of the pre-mRNA within the substructure as shown in Figure 7a. Therein, the initial and the last structure taken from trajectories are shown in red and blue, respectively. In the wild type system, both initial and last position of pre-mRNA and K700 residue are almost superimposed onto each other as shown in Figure 7a, whereas pre-mRNA deviated from its initial position in the mutant system as shown in Figure 7b. This dynamic change reflects the impact of the mutation on dynamics and orientation of the pre-mRNA within the SF3b complex.

## 3. Discussion

Identification of aberrant splicing, as one of the significant cancer drivers, has brought SF3B1 into prominence due to its crucial role in recognition of the proper branch sites during splicing process. The superhelical heat domain of SF3B1 is particularly critical for fulfilling its function and has been shown to be mutated in various cancer types. Therefore, there is an urgent need for understanding the impact of these mutations on structure and dynamics of splicing factor, SF3B1, which will help improve drug discovery studies that target mutant protein.

As a first step in that direction, we provided a mechanistic insight into the mechanism of altered branch point usage elicited by K700E mutation. We showed that the mutation affected interactions of pre-mRNA with SF3B1 and p14, which is in line with functional studies that demonstrate that point mutations in SF3B1 alter branch point selection [41] and also interactions between mutant SF3B1 and other spliceosome components [42,43].

Herein, it is important to emphasize that the altered interaction of p14 with pre-mRNA is novel and warrants follow-up *in vitro* models. Moreover, findings of dynamic cross correlation analysis provide an insight into the impact of mutation on interactions between SF3B1 and other proteins that are not part of the spliceosome. For instance, it has been shown that PPP1R8 interacts with SF3B1 through amino acids between 223-491 [44] and the cross correlation analysis showed that the interaction network among residues 455-832 changed upon mutation. Therefore, it is likely that the mutation distorts interaction also between SF3B1 and PPP1R8, thus leading to problems in (dis)assembly and catalytic steps of splicing. The information regarding network modulation can also be used to determine novel sites on SF3B1 to which small therapeutic molecules can bind and revert the mutant protein to its original function. Alternatively, this knowledge can also help improve existing drug discovery strategies that target mutant SF3B1 as in the case of riboside analog 8-azaguanine [45], H3B-8800 [46], and E7107 [47]. Among them, E7107 has been shown to enhance antitumor response when combined with the proteasome inhibitor, bortezomib, in triple-negative breast cancer, thus highlighting the possible usage of mutant SF3B1 inhibitors in combination therapies.

Our work, though need experimental validations, sheds light on the unknown mechanism of altered branch point usage caused by SF3B1-K700E mutation. Considering that the mutation is frequently seen in myelodysplastic syndrome (MDS), chronic lymphocytic leukemia (CLL) and breast cancer our results can be used to study other cancer types, and mutant SF3B1 can be considered a potential drug target for cancer therapy. Consequently, this demonstrates the general applicability of the study.

## 4. Materials and Methods

Previous studies showed that mutant genes, which are involved in cancer [48], encode abnormal proteins that interfere with various vital biological processes such as cell proliferation, transcription, splicing, immune response [49], cell communication, and apoptosis [50].Among them, aberrant splicing is considered one of the most significant driving factors for development and progression of cancer [51], as it leads to production of proteins with altered function. Therefore, “pre-mRNA splicing” is selected as the target biological process in this study.

As mentioned above, (SF3B1)${}^{K700E}$ mutant has been shown to be associated with various cancer types. As such, we wanted to investigate if it is among those having higher centrality and associated with the highest number of mutations in the gene network that is involved in pre-mRNA splicing before assessing the impact of the mutation *in silico*. To do so, we first identified the genes that participate in pre-mRNA splicing and then constructed the corresponding network as explained below.

### 4.1. Identification of Genes Participating in Pre-mRNA Splicing and Constructing the Related Gene Network

To ensure the general applicability of our findings around the mutation studied and to optimize the use of our limited computing resources, we created a generic pipeline to prioritize the genes/mutations that will be studied through molecular dynamics experiments. To this end, we devised a method that factors in two aspects; (1) the prevalence of the mutation in various cancer types, and (2) the centrality of the mutant gene in the target functional context, namely pre-mRNA splicing.

We first used Reactome database [52] to get the list of genes that are involved in mRNA splicing. First, we got 3734 genes which were decreased down to 86 upon filtering based on species as “homo sapiens” and the type as “protein”. STRING database [53] was used to construct a network of genes from these 86 proteins. It consisted of five connected components; four small isolated components consist of 5, 3, 3, and 2 nodes which are connected by 10, 2, 2, and 1 edges, respectively which is shown in Figure 1. The complete network has 67 nodes connected by 397 edges and 12 disconnected nodes (Figure S1), where the nodes represent genes and edges represent experimental interactions taken from different studies such as crystallography and purification assays, cross-linking, and expression level measurements.

We used NetworkAnalyzer plugin [54] within Cytoscape software platform [55] to compute topological parameters and centrality metrics for individual genes (i.e., nodes) on the constructed pre-mRNA splicing network, including closeness, radiality, and eccentricity. Thereafter, we also included somatic mutation frequency data coming from COSMIC database (September 2019 release, the latest version) [56] in our calculations to determine the gene with the highest number of mutations. COSMIC database was filtered based on the following criteria:

1. For practical reasons, we chose breast cancer, one of the most common cancer type, with large number of samples in COSMIC.
2. Only the histological type of “Carcinoma” was used as it is the most common histological type covering the majority of the breast malignancies [57,58].
3. Only “Substitution-Missense” somatic mutations are included. According to COSMIC statistics, 82% of breast carcinoma patients have somatic mutations of type “Substitution-Missense”.
4. Only somatic mutations that are predicted strictly as pathogenic mutations are included. Functional Analysis through Hidden Markov Models (FATHMM) is a predictive tool for the mutation’s impact and classifies any mutation as one of the following classes; pathogenic, neutral or not specified. In addition, FATHMM scores are given in the form of *p*-value that ranges from 0 to 1. By default, the pathogenic mutations have scores greater than 0.5. For our purposes, we further increased the threshold for the pathogenic mutations to 0.9 to avoid false positive predictions.

From the filtered somatic mutation dataset, we computed the number of mutations associated with each gene, and then we superimposed these data on the constructed pre-mRNA splicing. SF3B1 emerged a gene containing the highest number of mutations; K700E being the most frequent one, while having comparably high centrality in the constructed pre-mRNA splicing network to other genes as shown in Figure 1.

### 4.2. Assessment of the Impact of the Mutation by Means of Molecular Dynamics Simulations

In 2015, Maguire et al. analyzed available exome and whole genome sequencing data that belong to 1293 tumor samples and showed that SF3B1-K700E mutation appeared in 17 of 23 mutant samples [23]. Later, in 2018, Seiler et al. reanalyzed whole-exome sequencing data, which included 33 cancer types, to identify SF3B1-related somatic mutations. Consequently, a group of mutations was identified as hotspots regarding SF3B1, namely, R625C/H, E622D/Q, N626D/H/Y, K666E/N/T, K700E, G742D, L833F, E860K, and E862K [59]. Therefore, we utilized the most recent version of COSMIC database which includes almost all studies that report on the SF3B1-K700E mutant involved in breast cancer. In this way, we could i) identify the most frequent mutation among other hotspot mutations that are associated with the target gene in breast cancer and also ii) include a larger and more recent pool of samples. The results showed that K700E was detected in 45 samples while an unknown mutation was detected in 21 samples among 115 samples studied. Moreover, each of G83=/L982= (synonymous substitution), K666Q, R625C, and T935K mutations were detected only in 2 samples, and each of the remaining 44 mutations were detected in only one sample. Therefore, K700E substitution mutation (lysine ⇒ glutamic acid at position 700) was found to be the one with the highest frequency (40%) among other mutations associated with SF3B1. Consequently, K700E mutation was selected to be used in subsequent molecular dynamics (MD) simulations.

#### 4.2.1. System Setup

**Figure 8:**
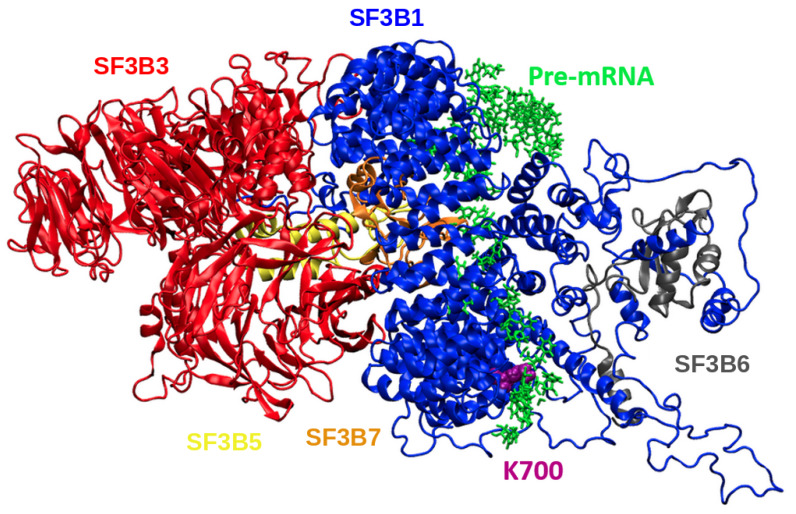
The depiction of the 3D structure of five domains of the SF3b complex included in this study. They are taken from the cryogenic electron microscopy (cryo-EM) structure of human spliceosome complex (PDB:5z56) [18]. Each domain is shown in a different color. SF3B1, SF3B3, SF3B5, SF3B6, SF3B7, and pre-mRNA are shown in blue, red, yellow, grey, orange, and green, respectively. The image was created using VMD [40].

Cryogenic electron microscopy (cryo-EM) structure of the human spliceosome (PDB ID:5Z56) at resolution 5.1 Å [18], which corresponded to B${}^{act}$ state, was retrieved from Protein Data Bank (PDB). The complex contains 52 proteins, three snRNAs, and synthetic pre-mRNA with a molecular weight of ca. 1.8 mega-Daltons. Due to the size of the system, we did not include all of SF3b complex’s components available in the cryo-EM structure, instead five chains, namely, SF3B1, SF3B3, SF3B5, SF3B6, SF3B7, and pre-mRNA (See Figure 8) were used in MD simulations since they directly interact with SF3B1. We used SWISS-MODEL web-server [60] to model the missing parts of the SF3B1 except the first 81 residues. We did not include both SF3B2 and SF3B4 in the final complex for the following reasons: i) they did not have any direct interactions with SF3B1, ii) SF3B2 has 895 residues but only 183 of them are available, and iii) SF3B4 has 424 residues, but only 78 residues are resolved in the experimental structure. We exploited CHARMM-GUI [61] to prepare both wild type and the mutant systems. CHARMM36 force field [62] was used to model protein and RNA, whereas water molecules were represented by TIP3P water model [63]. After including water molecules, wild type system has 1089028 while the mutant one has 1089281 atoms. The thickness of water layer was set to 16 Å in each direction to prevent interaction with the periodic images of the system. The system was neutralized with 0.15 M KCl.

#### 4.2.2. Simulation Protocol

Molecular dynamics simulations were performed by using GROningen MAchine for Chemical Simulations (GROMACS) [64] package. Equilibration of the system was performed by using the NVT ensemble, whereas the NPT ensemble was used in the production step to maintain constant pressure and temperature throughout the simulation. We used Nose-Hoover and Parrinello-Rahman coupling algorithms with 1 ps and 5 ps coupling times to maintain the temperature at 310 ${}^{\circ }$K and 1 bar, respectively. LINCS algorithm was used to constrain the bond lengths in hydrogen atoms [65]. The Particle Mesh Ewald (PME) method was used to compute long-range electrostatic interactions [66]. For van der Waals and short-range electrostatic interactions, we set the cut off value to (12 Å). The time step for integration was set to 2 femtoseconds. MD simulations were performed for 2 microseconds in total. Three replicates, each of which started with a different initial velocity distribution, were used for both wild type and the mutant system to check the reliability of the results. The two replicates were run for 500 nanoseconds (ns), whereas the last one was run for 100 ns. For the analyses, we used two trajectories each of which was around 500 ns.

#### 4.2.3. Simulation Analysis Tools

We used GROMACS package for MD trajectories analysis as it provides many tools and GRaphing and Advanced Computation and Exploration of data (Grace) tool for data plotting.

##### Root Mean Square Deviation (RMSD)

Root mean square deviation is the most commonly used similarity measurement tool [67] which is given by the following equation:

(1) $$\begin{array}{c} RMSD=\sqrt{\frac{1}{n}\sum_{i=1}^{n}d_{i}^{2}}, \end{array}$$

where *n* represents pairs of equivalent atoms and $d_{i}$ is the distance between the two atoms in the *i*th pair. Here, the RMSD is calculated by using the backbone atoms of the selected groups. Also, the initial structure in corresponding trajectories was used as the Reference [68].

##### Calculation of Number of Contacts

The number of contacts between pre-determined groups is computed by using “gmx mindist” module implemented in GROMACS. We used 0.3 nm as the cut-off value to calculate the number of contacts between the mutated residue and pre-mRNA while 3 nm was used to calculate the number of contacts between side chain residues of p14 (residues 20–100) and pre-mRNA as p14 is defined in previous studies as a marker of branch point [12,13].

##### Principal Component Analysis (PCA)

To determine the dominant motion of a structure, principal component analysis is computed by obtaining the covariance matrix of Cα atoms with respect to the reference structure. Covariance matrix is computed using “gmx covar” module in GROMACS.

(2) $$\begin{array}{c} C_{ij}=\langle M_{ij}\Delta r_{i}\Delta r_{j}\rangle , \end{array}$$

where $C_{ij}$ corresponds to covariance matrix of Cα atoms *i* and *j*. $M_{ij}\Delta r_{i}\Delta r_{j}$ refers to positional change from time-averaged structure for each coordinate of all Cα atoms *i* and *j*. Diagonalization of covariance matrix is performed using “gmx covar” module in GROMACS. It results in a set of eigenvalues $\delta^{2}$ and their corresponding eigenvectors (v).

(3) $$\begin{array}{c} \mathrm{Cv}=\delta^{2}v. \end{array}$$

We used “gmx anaeig” module to analyze the first two eigenvectors that represent the directions and relative magnitudes [69] of more than 50% of the dominant motion of the system. Additionally, we used “rmsf” module to plot root mean square fluctuation (RMSF) per atom of the first two eigenvectors. Therefore, we can identify a particular residue (or a set of residues) that leads to fluctuation of the overall chain. RMSF is given as RMSD from the average over time calculated per each residue and with respect to the initial frame [64]. RMSF is expressed as

(4) $$\begin{array}{c} RMSF=\sqrt{\frac{1}{N}\sum_{n=1}^{N}(X_{i}\left(n\right)-{\bar{X}}_{i})}, \end{array}$$

where *N* corresponds to the number of frames obtained from simulation, $X_{i}\left(n\right)$ corresponds to the coordinates of backbone atom of $X_{i}$ and ${\bar{X}}_{i}$ is given as the average coordinate obtained from simulation.

##### Dynamic Cross Correlation Map (DCCM)

Dynamic cross correlation (DCC) among Cα of SF3B1 residues is computed using Bio3d package for biological structural analysis written in R programming language [70]. Bio3d library contains major functions needed for structural bioinformatics. For this study, we used “dccm.xyz” and “plot.dccm” modules to generate dynamical cross correlation matrices and to plot them, respectively. DCC is expressed as

(5) $$\begin{array}{c} DCC\left(i,j\right)=\frac{<\Delta r_{i}\left(t\right).\Delta r_{j}\left(t\right){>}_{t}}{\sqrt{<{\|\Delta r_{i}\left(t\right)\|}^{2}{>}_{t}}\sqrt{<{\|\Delta r_{j}\left(t\right)\|}^{2}{>}_{t}}}, \end{array}$$

where $r_{i}\left(t\right)$ and $r_{j}\left(t\right)$ correspond to the coordinates of the *i*th and *j*th atoms as a function of time *t*, <.> indicates the time ensemble average, $\Delta r_{i}\left(t\right)=r_{i}\left(t\right)-{\left(<r_{i}\left(t\right)>\right)}_{t}$ and $\Delta r_{j}\left(t\right)=r_{j}\left(t\right)-{\left(<r_{j}\left(t\right)>\right)}_{t}$ [71,72,73].

## Supplementary Materials

The following are available online at https://www.mdpi.com/2218-273X/10/5/680/s1, Figure S1: Depiction of the components of the constructed gene network that contains genes participating in mRNA splicing mechanism.
