# Supplementary material for: In Silico Analysis of a Highly Mutated Gene in Cancer Provides Insight into Abnormal mRNA Splicing: Splicing Factor 3B Subunit 1K700E Mutant
Source: Biomolecules. 2020 Apr 28;10(5):680. doi: 10.3390/biom10050680 (PMC7277358; doi:10.3390/biom10050680)
Supplement: Supplementary file 1 [file biomolecules-10-00680-s001.zip › SupplementaryFiles/Supplementary Information_updated.docx]

**Supplementary Information**

**In Silico Analysis of a Highly Mutated Gene in Cancer Provides Insight Into Abnormal mRNA Splicing: Splicing Factor 3B Subunit 1^K700E^ Mutant**

**Asmaa Samy^1^, Baris Ethem Suzek^2^, Mehmet Kemal Ozdemir^1^ and Ozge Sensoy^1,3,*^**

^1^ The Graduate School of Engineering and Natural Science, Istanbul Medipol University, Istanbul, Turkey.

^2^ Department of Computer Engineering, Mugla Sıtkı Koçman University, Mugla, Turkey.

^3^ Regenerative and Restorative Medicine Research Center (REMER), Istanbul Medipol University, Istanbul, Turkey.

* Correspondence: osensoy@medipol.edu.tr

**Supplementary Figure**


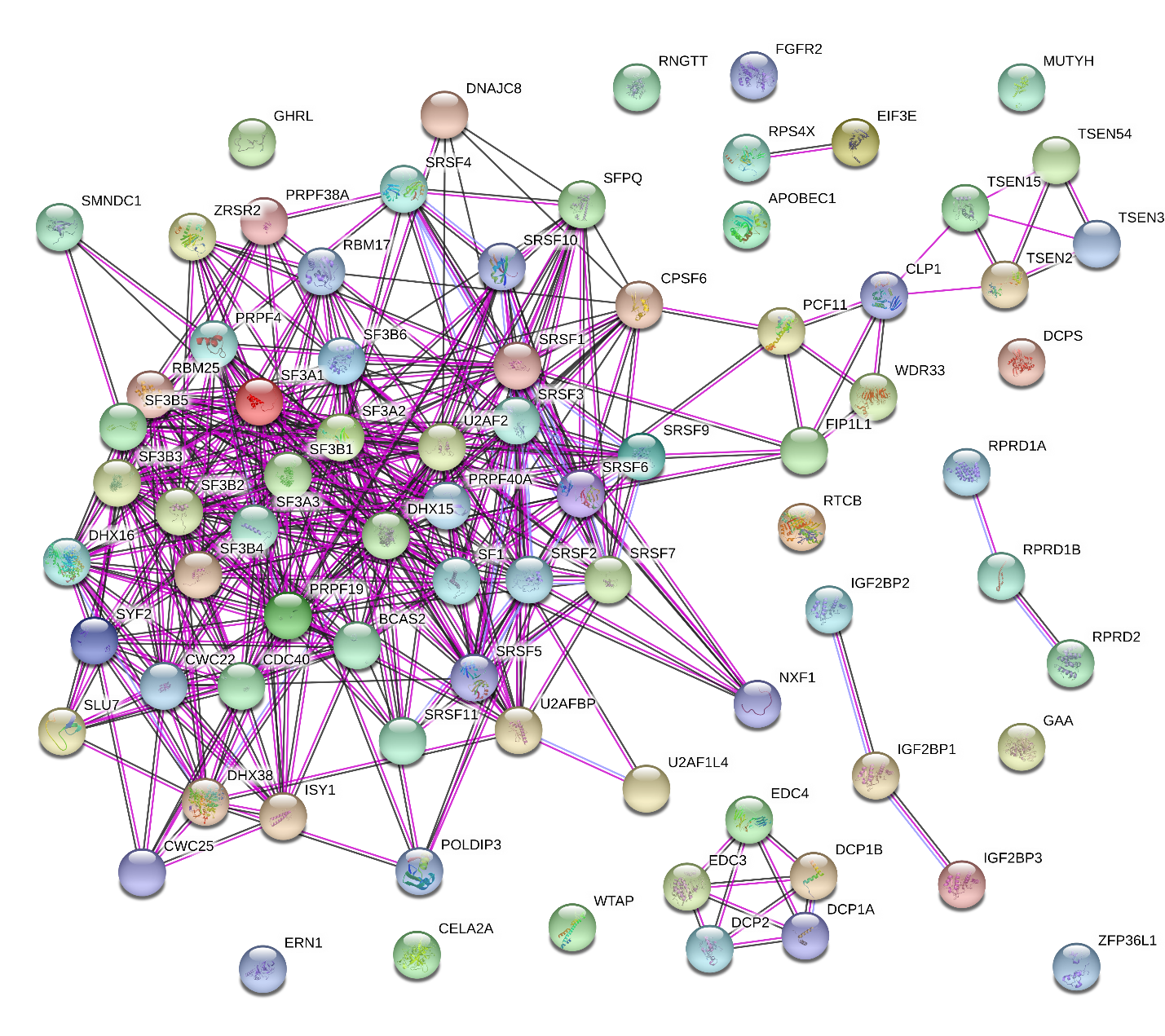


**Figure S1**. Depiction of the components of the constructed network. Overall, the network has 67 nodes connected by 397 edges and 12 disconnected nodes. 67 nodes compose five connected components. The giant component of the network consists of 54 nodes and 382 edges. Besides, four small isolated components consist of 5, 3, 3, and 2 nodes which are connected by 10, 2, 2, and 1 edge, respectively.
